# The secondary KIT mutation p.Ala510Val in a cutaneous mast cell tumour carrying the activating mutation p.Asn508Ile confers resistance to masitinib in dogs

**DOI:** 10.1186/s12917-020-02284-9

**Published:** 2020-02-19

**Authors:** Fabio Gentilini, Maria Elena Turba, Claire Dally, Masamine Takanosu, Sena Kurita, Makoto Bonkobara

**Affiliations:** 1 Department of Veterinary Medical Sciences, University of Bologna, via Tolara di sopra 50, 40064 Ozzano dell’Emilia, BO Italy; 2 Genefast srl, Via Jolanda Baldassari 6, 47122 Forlì, Italy; 3 Laboratoire d’Anatomie Pathologique Vétérinaire du Sud-Ouest, 129, Route de Blagnac, 31201 Toulouse cedex 2, France; 4 Nasunogahara Animal Clinic 2-3574-98, Asaka, Ohtawara, Tochigi 324-0043 Japan; 5 Department of Veterinary Clinical Pathology, Nippon Veterinary and Life Science University, 1-7-1 Kyonan-cho, Musashino-shi, Tokyo, 180-8602 Japan

**Keywords:** Dogs, KIT, Mast cell tumours, Resistance, Somatic mutation

## Abstract

**Background:**

Gain-of-function mutations in KIT are driver events of oncogenesis in mast cell tumours (MCTs) affecting companion animals. Somatic mutations of KIT determine the constitutive activation of the tyrosine kinase receptor leading to a worse prognosis and a shorter survival time than MCTs harbouring wild-type KIT. However, canine MCTs carrying KIT somatic mutations generally respond well to tyrosine kinase inhibitors; hence their presence represents a predictor of treatment effectiveness, and its detection allows implementing a stratified medical approach. Despite this, veterinary oncologists experience treatment failures, even with targeted therapies whose cause cannot be elucidated. The first case of an MCT-affected dog caused by a secondary mutation in the tyrosine kinase domain responsible for resistance has recently been reported. The knowledge of this and all the other mutations responsible for resistance would allow the effective bedside implementation of a deeply stratified and more effective medical approach.

**Case presentation:**

The second case of a canine MCT carrying a different resistance mutation is herein described. The case was characterised by aggressive behaviour and early metastasis unresponsive to both vinblastine- and masitinib-based treatments. Molecular profiling of the tumoural masses revealed two different mutations; other than the already known activating mutation p.Asn508Ile in *KIT* exon 9, which is tyrosine kinase inhibitor-sensitive, a nearly adjacent secondary missense mutation, p.Ala510Val, which had never before been described, was detected. In vitro transfection experiments showed that the secondary mutation did not cause the constitutive activation by itself but played a role in conferring resistance to masitinib.

**Conclusions:**

This study highlighted the importance of the accurate molecular profiling of an MCT in order to improve understanding of the molecular mechanism underlying tumourigenesis and reveal chemoresistance in MCTs for more effective therapies. The detection of the somatic mutations responsible for resistance should be included in the molecular screening of MCTs, and a systematic analysis of all the cases characterised by unexpected refractoriness to therapies should be investigated in depth at both the genetic and the phenotypic level.

## Background

Gain-of-function mutations in *KIT* are driver events of oncogenesis in mast cell tumours (MCTs) and gastrointestinal stromal tumours (GISTs) affecting companion animals [1–8]. Evidence has been accumulating which has demonstrated that acquired somatic mutations of *KIT* determine the constitutive activation of the tyrosine kinase receptor, causing a worse prognosis and a shorter survival time than MCTs harbouring wild-type KIT [9–14]. Activating mutations in canine MCTs have been reported in exons 8 and 9 (encompassing the outer immunoglobulin-like domains which interact with the stem cell factor [SCF] ligand), in exon 11 (encompassing the juxtamembrane domain involved in the signal transduction) and in exon 17 (which codes a tyrosine kinase domain) [1, 2, 5, 7, 12].

Imatinib mesylate is the archetypal molecule of class III tyrosine kinase inhibitors (TKIs), receiving market approval for the treatment of certain types of haematological malignancies carrying *KIT* mutations in humans. Since its approval, imatinib has changed the paradigm of cancer treatment due to its outstanding efficacy and relative safety. However, after a few years of worldwide use, it became increasingly evident that the use of imatinib over time was associated with the occurrence of secondary resistance [15]. The mechanisms of secondary resistance against TKIs encompass point mutations in the kinase domains, gene amplification and/or overexpression, overproduction of P-glycoprotein, inhibition of the transporter responsible for the uptake of imatinib into cells, and constitutive activation of the downstream signal transduction of receptor tyrosine kinases [16–21]. Of these, point mutations occurring in the kinase domains are the most frequently reported in humans; the latter evidence, i.e. mutations in exons 14 and 17, has also been described in cell lines derived from canine MCTs and, recently, in an MCT-affected dog [22–24].

The case of a French bulldog affected by an aggressive and unresponsive form of MCT carrying the activating mutation of exon 9, p.Asn508Ile (c.1523A > T), and a secondary mutation very close to the previous one, KIT p.Ala510Val (c.1529C > T) which conferred resistance to masitinib is herein reported for the first time.

## Case presentation

### Ethical statement

This study did not require official or institutional ethical approval as it was not experimental. The animals were handled according to good ethical standards and European Union legislation. The dog in this study was examined with the written consent of their owner. The aim was to identify the cause of the disease and thereby improve the animal welfare.

### Case description

A 6 year-old, intact male French bulldog was examined by its veterinary practitioner for the presence of two rapidly growing subcutaneous masses in the lumbar region. During the clinical examination, abdominal palpation revealed a large abdominal mass. Ultrasound imaging confirmed the presence of a large mass arising from the left kidney. The two cutaneous masses were surgically removed by conventional surgery. The renal mass was biopsied with an 18 Gauge sheathed needle (Tru-Cut) / automatic biopsy gun under real-time ultrasound guidance. A high-grade mast cell tumour with renal metastasis was diagnosed, and treatment with vinblastine and cortisone was promptly instituted. In the meantime, the cutaneous samples also underwent KIT mutation analysis. Eventually, the findings of the activating mutation p.Asn508Ile supported the decision to abandon the current treatment with vinblastine, which had not shown any improvement, and to start treatment with masitinib (Masivet, [AB Science] 150 mg, 12.5 mg/kg, once daily). Regrettably, no effective reduction in the masses was noted. One month later, the dog’s clinical condition worsened and the owners elected for humane euthanasia. Necropsy was declined by the owners.

### Histology and immunohistochemical staining

Formalin-fixed samples underwent histological and immunohistochemical examination. Paraffin sections of each sample were stained with hematoxylin and eosin (HE) and toluidine blue (tolonium chloride), and were immunolabeled using antibodies specific for the detection of the CD117 antigen coded by the *KIT* gene according to standard protocols. Immunolabeling was carried out retrospectively and was monitored by an external control represented by a normal dog skin sample.

### Mutational analysis

#### Genomic DNA purification

The genomic DNA (gDNA) was purified from formalin-fixed paraffin-embedded (FFPE) tissue specimens of both cutaneous samples and renal masses using a Maxwell® FFPE Plus DNA Kit run on the Maxwell® RSC 48 Instrument (Promega, Milan, Italy) according to the manufacturer’s instructions. The renal sample was examined retrospectively.

#### PCR

Briefly, the polymerase chain reaction (PCR) protocols for amplifying exon 9 consisted of Phusion polymerase (Thermo Scientific) 0.4u, HF buffer 1X, deoxyribonucleotide triphosphates (dNTPs) 200 μM each, additional Mg 0.25 mM, 500 nM each forward (5′ ACTCGTCTCTGTCACCGTCTGGAA 3′) and reverse (5’ATGGCAGGCAGAGCCTAAACATCC 3′) primers, 1 μL of template DNA and water molecular grade reagent up to 25 μL. The amplification program consisted of 98 °C for 30 s, followed by 40 cycles involving denaturation at 98 °C for 10 s, annealing at 69 °C for 10s and extension at 72 °C for 10 s.

#### DHPLC and sequencing

The *KIT* Exon 9 PCR amplicon was run on an automated denaturing high-performance liquid chromotography (DHPLC) apparatus (WAVE DHPLC system, ADS Biotec Limited) equipped with a proprietary column (DNASep, Transgenenomic). Elutions were carried out with a mixture of Buffer A (0.1 mol/L triethylammonium acetate–TEAA) and Buffer B (0.1 mol/L TEAA, 25% acetonitrile). The amplicons were run at two different partially-denaturing temperatures of 56.3 °C and 57.8 °C used for the DHPLC screening of the entire exon 9, including the p.Asp508Ile mutation as previously reported [25]. The data analysis was carried out using Navigator software, (ADS Biotec Limited). The PCR products were then purified using ExoSAP-IT PCR Product Clean-Up kit, and forward and reverse direct sequenced using Big-Dye terminator chemistry, additionally purified with Centri-Sep columns (Life Technologies, Monza, Italy) and electrophoresed on an ABI Prism 310 automated sequencer.

#### Cell line

The cell line, HEK293 cells which are human embryonic kidney cells which are easy to transfect, was kindly provided by Dr. Tanaka, Nippon Veterinary and Life Science University, Japan. The HEK293 cells were maintained in complete Dulbecco’s modified Eagle’s medium (cDMEM) (Life Technologies, Monza, Italy) supplemented with 10% fetal calf serum (Nippon Bio-Supply), 50 U/mL penicillin (Life Technologies, Monza, Italy), and 50 μg/mL streptomycin (Life Technologies, Monza, Italy) in a humidified incubator at 37 °C under 5% CO2.

#### Analysis of the phosphorylation status of KIT and mutant KIT in HEK293 cells

The phosphorylation status of both KIT and mutant KIT were analysed as described previously [24].

Briefly, using mammalian expression vector pcDNA3.1 containing wild-type KIT derived from normal canine peripheral blood mononuclear cells, the variations c.1523A > T and/or c.1529C > T were inserted using a site-directed mutagenesis kit (PrimeSTAR Mutagenesis Basal Kit, Takara). The HEK293 cells suspended in cDMEM were plated in a six-well plate and cultured for 24 h. The KIT expression vectors encoding KIT carrying c.1523A > T, c.1529C > T or c.1523A > T plus c.1529C > T mutations were then transiently transfected into the HEK293 cells using polyethylenimine MAX (Polyscienses) according to the manufacturer’s protocol. After transfection, the HEK293 cells were cultured for another 8 h and were then serum starved for 16 h. The HEK293 cells were then additionally cultured with masitinib in order to study the tyrosine kinase inhibition (ChemScene; 0–10 μM) for 90 min. The cells were then lysed, and the aliquots underwent Western blotting using the following antibodies: polyclonal rabbit anti-human CD117 antibody (Dako) or monoclonal rabbit anti-human phosho-c-Kit (Tyr703) antibody (Cell Signaling), followed by horseradish peroxidase-conjugated donkey anti-rabbit immunoglobulin G whole antibody (GE Healthcare). The immunoreactive bands were visualised using an enhanced chemiluminescence system (GE Healthcare) and the LAS-4000 (Fujifilm). The band intensities were semi-quantified using ImageQuant TL software (Fujifilm), and signal levels for phosphorylated KIT were normalised to the levels of KIT expression.

## Discussion and conclusions

**Fig. 1:**
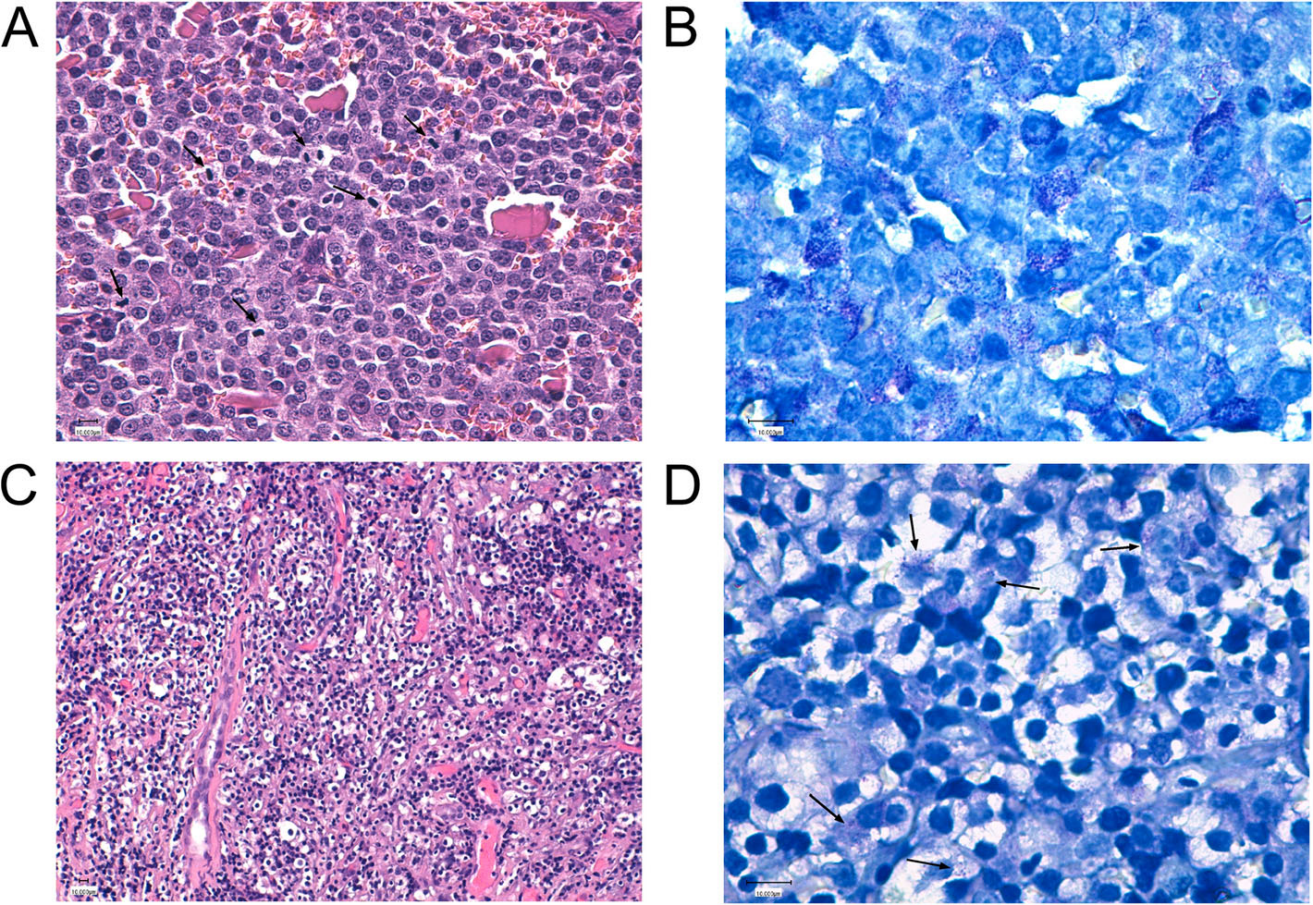
**a** Cutaneous mass HE × 200 Neoplastic round cells with numerous mitoses (arrows); **b**) Cutaneous mass Toluidine Blue × 400: numerous metachromatic granules in the cell cytoplasm; **c**) Renal biopsy: HEx200: Neoplastic round cells replacing the renal parenchyma (one residual tubule still visible); **d**) Renal biopsy: Toluidine Blue × 400: Regular inconspicuous metachromatic granulations (arrows)

In stratified (also referred to as individualised or precision) medicine, a precise tailored characterisation of tumours is essential for accurate patient management. A case of aggressive and rapidly fatal cutaneous MCTs with renal metastasis was herein reported. Indeed, histological examination of the cutaneous masses revealed that they were composed of infiltrating dermo-hypodermal sheets of neoplastic cells mixed with eosinophils. The cells were well-delineated round cells with moderately abundant and slightly granular cytoplasm. The nuclei were round and central with one or two small nucleoli. Anisocytosis and anisocaryosis were notable, and the mitotic index was greater than 8 mitoses in 10 high power fields (Fig. 1a). Using toluidine blue staining, the neoplastic cell cytoplasm revealed large number of metachromatic granules (Fig. 1b). All these findings supported a diagnosis of two high-grade cutaneous mast cell tumours according to the Kiupel grading system [11]. Both cutaneous tumours showed similar profiles with cytoplasmic paranuclear staining and increased membranous staining in the majority of the neoplastic cells as compared to normal mast cells. In particular, the cytoplasmic localisation was rarely diffused and was most often in the form of a large paranuclear granular marking spot of reinforced intensity [4, 26, 27]. In the biopsies of the kidney mass, the tubules were atrophic, and the interstitium was markedly enlarged by fibrosis, and a mixed neutrophilic and lymphoplasmacytic infiltrate. In one sample, the interstitium was infiltrated by sheets of poorly defined round cells with pale slightly granular cytoplasm, marked cytonuclear atypia and some mitotic figures (Fig. 1c). Toluidine blue staining revealed inconspicuous but regular metachromatic granules in the cytoplasm of these cells, supporting the diagnosis of a metastatic mast cell tumour (Fig. 1d). Renal biopsy did not show mast cell infiltration significant enough to be evaluated with immunohistochemistry.

High-grade MCTs respond variably to therapy, even if overall survival and progression-free survival are shorter than in low-grade MCTs [9, 11–14]. Furthermore, high-grade MCTs or those with KIT protein aberrant localisation (aberrant KIT protein localisation; KIT staining pattern II or III) are more likely to carry KIT mutations with respect to low-grade mutations [27]. As for humans, KIT mutation-driven MCTs have a worse prognosis, although they respond to TKIs better than wild-type MCT [4, 7, 11, 28, 29].

**Fig. 2:**
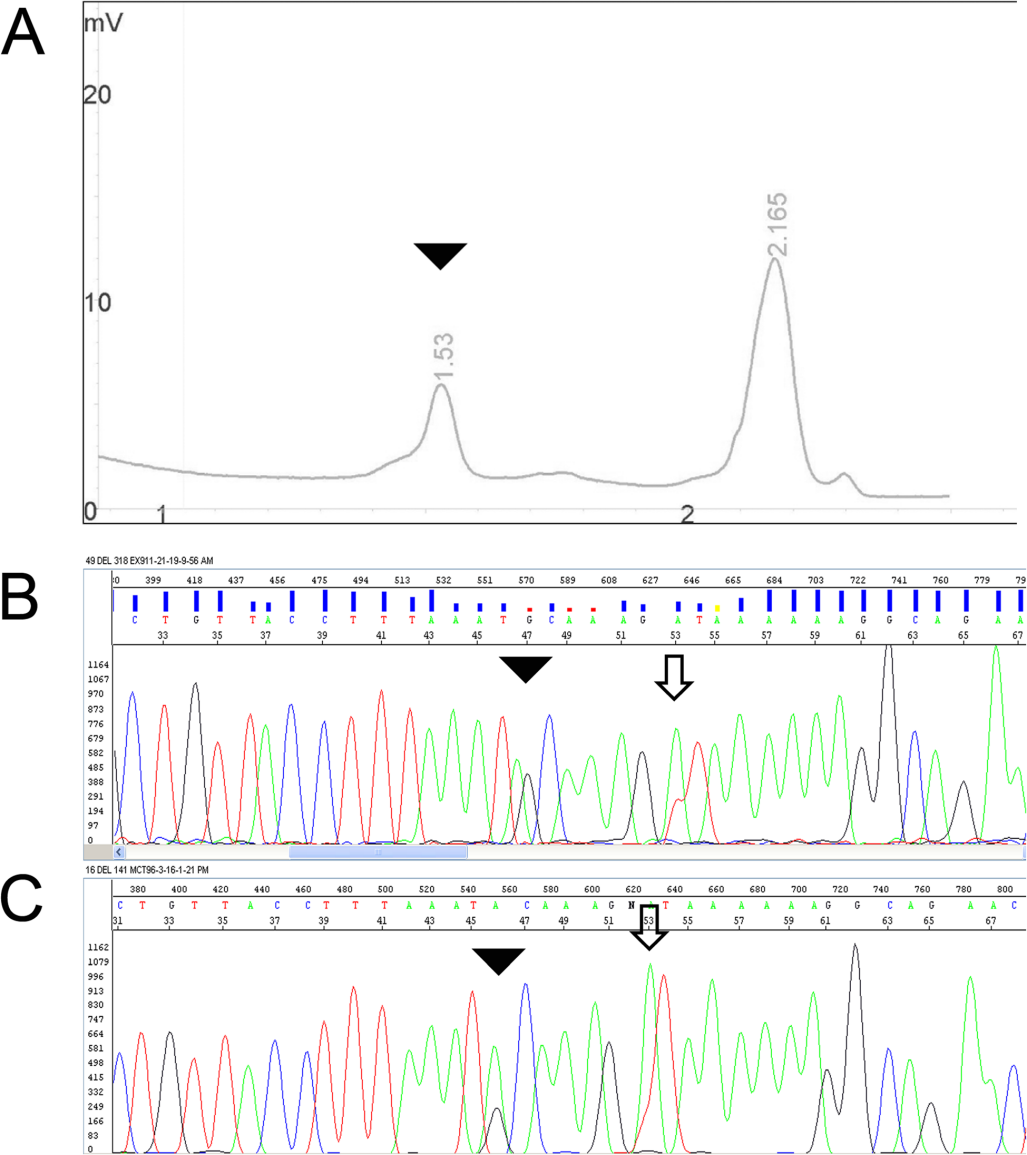
**a** Denaturing high-performance liquid chromotography (DHPLC) chromatograms: the peak profile of the case presented herein; the peak with a retention time of 1.53 min. (black arrow) indicates the presence of a heteroduplex caused by the presence of the mutation (c.1529C > T). the x-axis indicates DHPLC elution time in minutes and the y-axis the DNA concentration as measured by optical density at 260 nm in arbitrary units. A: **b**) Sanger sequencing chromatogram from the reverse primer of the renal sample and **c**) of the cutaneous samples, both indicating the presence of the primary (c.1523A > T; empty arrow) and secondary (c.1529C > T; plain arrowhead) mutations. The different heights of the peaks are evident. Likewise, the same pattern of mutations indicates the clonal origin of the primary lesion and distant metastasis

To refine the prognosis and to support TKI therapy, the MCT was investigated for *KIT* somatic mutations in exons 8,9,11, 14 and 17 using high sensitivity DHPLC mutational screening and a Genescanning assay followed by direct sanger sequencing [24]. To date, almost all activating somatic mutations in dogs have been described in these exons. Furthermore, the mutation responsible for resistance to the TKIs reported up to now [23] was also included in the analysis. The DHPLC screening detected mutations in exon 9 (Fig. 2a). The amplicon was sequenced using Sanger sequencing. Two mutations, i.e. p.Asn508Ile (c.1523A > T) and/or p.Ala510Val (c.1529C > T), were identified, the former being highly prevalent and the latter less prevalent than the previous one, based on the height of the corresponding peaks in sequencing chromatograms (Fig. 2b; Supp. Figure S1). The same finding was also identified in the renal sample demonstrating the clonal origin of the distant masses [30], interestingly signifying that the metastasis had occurred after the primary mass had already acquired the secondary mutation. Thereafter, the p.Asn508Ile (c.1523A > T) will be referred to as the primary mutation and p.Ala510Val (c.1529C > T) as the secondary mutation. The p.Asn508Ile has already been demonstrated to be an activating mutation, and MCTs carrying this mutation respond well to TKIs (imatinib) [10, 22, 31]. In this case, its presence clearly conflicted with the course of the disease and treatment response. An ongoing inherent resistance mechanism was hypothesised and investigated. Conversely, the missense mutation p.Ala510Val (c.1529C > T) has never been described until now. Thus the secondary mutation was additinally investigated, transfecting cell lines with expression vectors containing the wild-type or the variants [22].

**Fig. 3:**
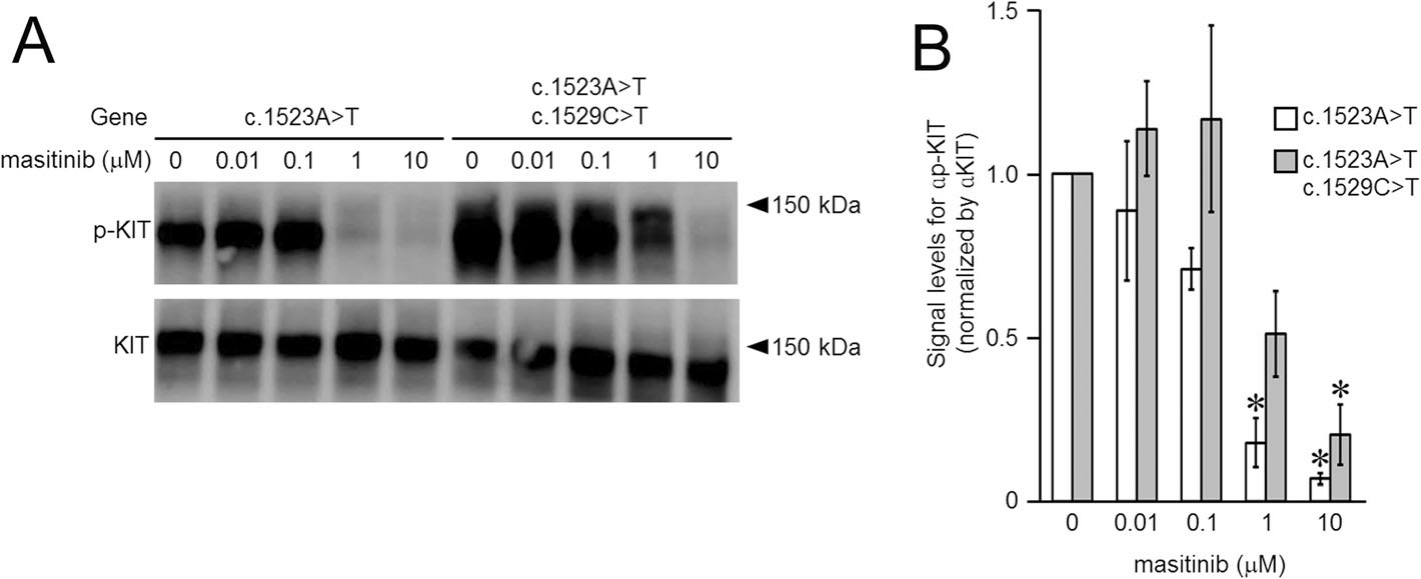
Western blot analysis: **a**) western blot appearance and **b**) semi-quantified data graphs of A (*n* = 3; three independent experiments). Phosphorylation of the dual mutant KIT was only suppressed at a masitinib concentration of 10 μM. * indicates significant difference (*P* < 0.01; Student *t*-test)

The KIT protein bearing the c.1523A > T mutation alone or along with the c.1529C > T mutation was found to display kinase activity, independent of ligand binding, as indicated by the phospho-KIT bands in Fig. 3. In addition, while phosphorylation of the mutant KIT with the primary mutation (c.1523A > T) was progressively suppressed with increasing concentrations of masitinib, and strong inhibition was observed at masitinib concentrations of 1 and 10 μM, the kinase activity of the double mutant was only suppressed at a masitinib concentration of 10 μM (Fig. 3). These findings revealed that the secondary mutation (c.1529C > T) conferred masitinib insensitivity to KIT. In fact, the maximum concentration (C_max_) of masitinib in dogs is 1.3–1.5 μM when given at an oral dose of 10 mg/kg, which is close to a clinical dose of 12.5 mg/kg [32]. The dose range setting (0, 0.1, 1, and 10 μM) used in this study covered plasma-achievable concentrations of masitinib in dogs. It is unlikely that a higher dose could have overcome the resistance but worth investigating further. Unfortunately, the dog in this study did not respond at all, when using either vinblastine or masitinib.

In humans, the majority of the kinase inhibitor-resistant mutations in receptor tyrosine kinases are located at the intracellular domain (e.g., KIT Asp816, PDGFR Asp842 or gate-keeper mutations). However, mutations in the extracellular domain may also cause resistance to kinase inhibitors (e.g., PDGFRA Tyr288Cys) [33]. In canine MCTs, the occurrence of secondary mutations responsible for TKI resistance following continuous exposure to TKIs has also been documented as a proof of concept in vitro and then also in vivo in a case of an MCT [22–24]. In particular, two studies have evaluated an MCT-derived cell line carrying the p.Asn508Ile mutation (c.1523A > T) which gave rise to an imatinib-resistant or toceranib-resistant subline carrying a secondary mutation in exon 14 (tyrosine kinase domain 1) at position Asn679Leu and in exon 17 (tyrosine kinase domain 2) at positions Asp815 (p.Asp815His, c.2443G > C), Asp819 (p. Asp819Val, c.2456A > T; p. Asp819Gly, c.2456A > G) of the protein [22, 24]. Furthermore, the first reported clinical case of resistance to imatinib carried an activating mutation in exon 11 and a secondary mutation c.2006C > T (p.Thr669Ile) in exon 14 which codes for tyrosine kinase domain 1 [23].

Toceranib is an alternative TKI approved for the treatment of MCT in dogs. Usually, MCTs with a KIT gene mutation respond well with an objective response rate of 60.0% [34] and it has recently been demonstrated that c.1523A > T mutation-driven constitutively activated MCTs are sensitive to toceranib [24]. Unfortunately, the dog was not treated with this TKI and the assessment of the full resistance pattern conferred by the secondary mutation herein described was beyond the aim of the study. However, the possibility that KIT resistance mutations might have a different rersistance pattern in different TKIs should be addressed in further studies. In this respect, addressing this issue could be of great aid in implementing strategies for targeting pre-existing resistant mutants or for preventing the development of resistance via genetic evolution.

In this study, it has additionally been confirmed that the existence of secondary somatic mutations of *KIT* in canine MCTs, is an ongoing mechanism of resistance to TKIs and that, more broadly, the mutations responsible for resistance could also occur in the extracellular domains of KIT. The mutations in the extracellular domains of KIT are driver- and kinase inhibitor-sensitive; however, this report demonstrated that this is not always true, and that, when using TKIs, the need to focus on the property of individual mutations rather than on the position of the mutation is warranted.

Unfortunately, the course of the disease was so rapid and the MCT so unresponsive that paired samples pre and post treatment could not be examined. In fact, the two mutations were found in the pre-therapy samples. Thus, it seemed that the somatic mutations were both pre-existing and not induced by the treatments, In fact, there may be several mechanisms for acquiring resistance by means of somatic mutations. It is a matter of controversy whether secondary mutations responsible for resistance pre-exist at very low levels and emerge as a consequence of the treatment [35–37] or whether they are induced by the treatment itself [38]. In veterinary medicine, Kobayasi et al. [22] and Kurita et al. [24] have demonstrated that prolonged exposure to TKIs induced the emergence of resistance by means of somatic mutations. Notably, it was herein shown that, in advanced highy aggressive tumours, somatic mutations responsible for resistance may already be present and have emerged before treatment.

Somatic mutations in *KIT* are usually found in a heterozygous state characterised by overlapping mixed peaks at a 1:1 ratio or less, depending on the frequency of the mutation or the number of normal cells infiltrating the tumour, among other factors. On the contrary, it is very rare to find somatic mutations almost entirely depicting the peak in the chromatogram which could only be interpreted as a homozygous or hemizygous variation [39, 40]. Notably, the p.Asn508Ile (c.1523A > T) variation had previously been demonstrated to ensue as a single allele [22] whereas, in the case herein reported, the almost complete substitution shown by the features of the c.1523A > T chromatogram peak supported the hypothesis that the variation occurred in homozygosis (two mutated alleles) or in hemizygosis (one mutated allele and one lost allele). These findings could be interpreted as secondary genetic instability events associated with the derangement of the DNA maintenance machinery in advanced neoplasia. In canine MCTs, very scarce information is available regarding zygosity in *KIT* mutations. However, in human GISTs, the topic of *KIT* zygosity has been investigated [39–44]. The hemizygous and homozygous KIT mutations were found to represent the minority of tumours, although the homo/hemizygosity was strongly associated with a malignant course and with metastatic disease [39–42]. In this case, the malignant course and metastatic disease could be explained by the presence of p.Asn508Ile in a homo- or a hemizygous state. A main drawback was that Sanger sequencing could not be used to ascertain the zygosity in cancer, and more accurate and reliable methods are warranted to aditionally confirm the prognostic importance of zygosity assessment in canine MCTs.

This case illustrated an archetypal situation for genotype driven targeted therapy. In fact, not all TKIs are equivalent or overseeded by the same resistance mutation and there is much evidence that different TKI molecules have a specific effectiveness profile [29, 31, 45, 46]. The tumour response to masitinib depends on the mutational profile of the individual tumour which may affect other TKIs differently [7, 29] e.g., the primary and secondary resistance profile may differ. In vitro toceranib was shown to favour the occurrence of resistance throughout the acquisition of secondary mutations involving the KIT tyrosine kinase domains [24]; no evidence exists that mutations in the extracellular domains may confer resistance to toceranib. Unfortunately, with few exceptions, these traits are not well defined in veterinary medicine. An unrivalled effort to better understand the peculiar features of each MCT in order to tailor individualised therapy represents the challenge for the future.

## Supplementary information

**Additional file 1.** Schematic representation of the *KIT* exon 9 mutations p.Asn508Ile (c.1523A>T) and p.Ala510Val (c.1529C>T). Both wild type (upper) and mutated (bottom) nucleotide and amino-acidic sequences are indicated.

## Data Availability

The datasets used and/or analysed during the current study are available from the corresponding author by reasonable request.

## Abbreviations

cDMEM: Complete Dulbecco’s modified Eagle’s medium
DHPLC: Denaturing high-performance liquid chromotography
dNTPs: Deoxyribonucleotide triphosphates
GISTs: Gastrointestinal stromal tumours
HE: Hematoxylin and eosin
MCT: Mast cell tumours
PCR: Polymerase chain reaction
SCF: Stem cell factor
TKI: Tyrosine kinase inhibitors
