# Supplementary figures and images for: The secondary KIT mutation p.Ala510Val in a cutaneous mast cell tumour carrying the activating mutation p.Asn508Ile confers resistance to masitinib in dogs
Source: BMC Vet Res. 2020 Feb 19;16:64. doi: 10.1186/s12917-020-02284-9 (PMC7029481; doi:10.1186/s12917-020-02284-9)

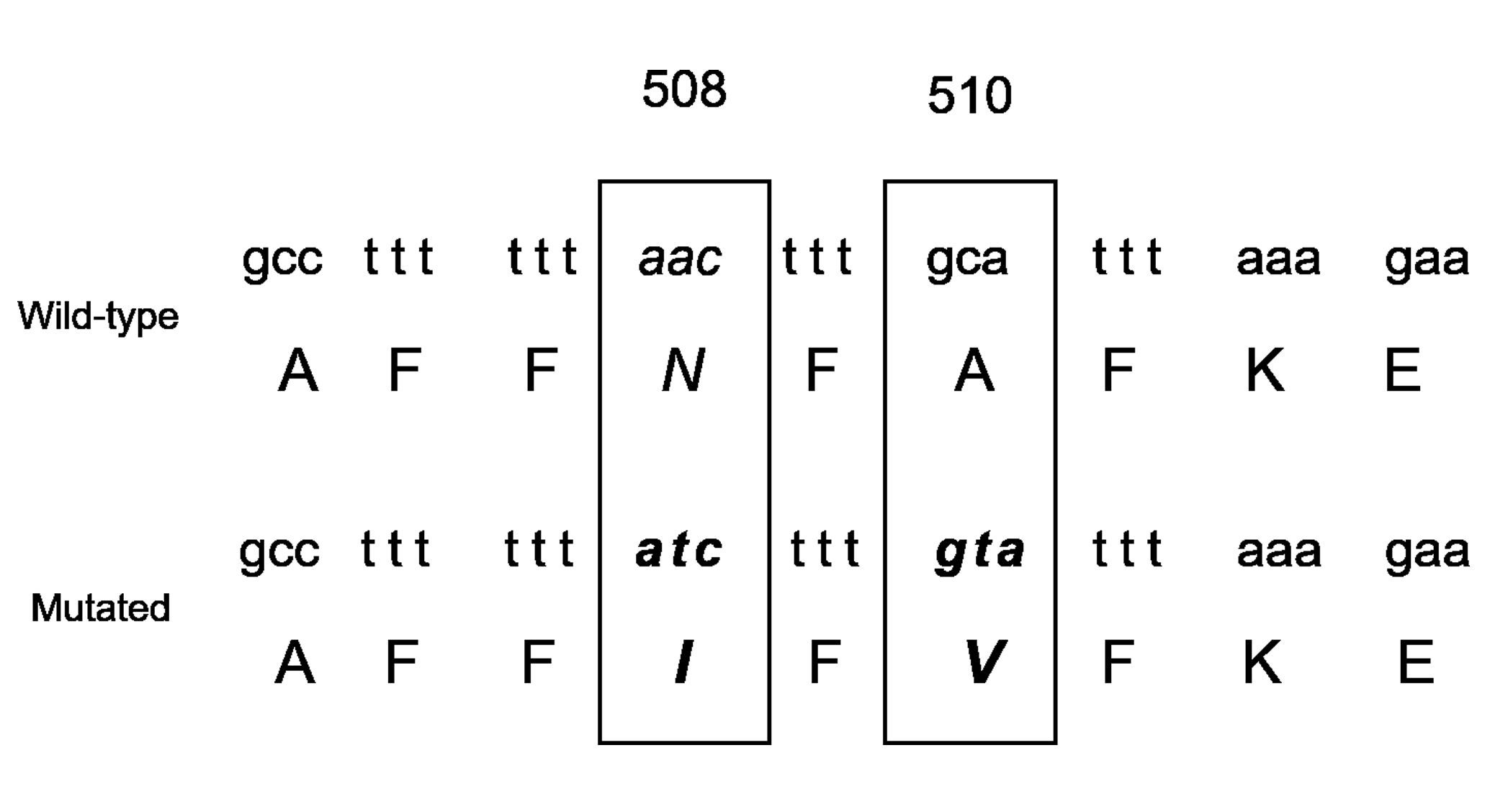

Supplement: Supplementary file 1 — Additional file 1. Schematic representation of the KIT exon 9 mutations p.Asn508Ile (c.1523A>T) and p.Ala510Val (c.1529C>T). Both wild type (upper) and mutated (bottom) nucleotide and amino-acidic sequences are indicated. [file 12917_2020_2284_MOESM1_ESM.tif]
